# Editorial: Novel Cancer Treatments Based on Autophagy Modulation

**DOI:** 10.3389/fphar.2021.650559

**Published:** 2021-04-16

**Authors:** Marco Cordani, Álvaro Somoza, Marco Tafani, Ilaria Dando, Suresh Kumar

**Affiliations:** ^1^ IMDEA Nanociencia, Ciudad Universitaria de Cantoblanco, Madrid, Spain; ^2^ Department of Experimental Medicine, Sapienza University of Rome, Rome, Italy; ^3^ Department of Neurosciences, Biomedicine and Movement Sciences, Section of Biochemistry, University of Verona, Verona, Italy; ^4^ Department of Molecular Genetics and Microbiology, University of New Mexico Health Sciences Center, Albuquerque, NM, United States

**Keywords:** cancer treatment, autophagy, pharmacology, nanomedicine, chemoresistance, signalling pathways

Autophagy is an intracellular process in response to stimulus by which damaged organelles, protein aggregates, or invading pathogens, are targeted from autophagic vesicles to lysosomes and then eliminated (Mizushima et al., 2011). Autophagy has been described to play a role in several pathological conditions, including neurodegenerative disorders, infectious diseases, and cancer.

The role of autophagy in cancer remains highly controversial, and it is likely reliant on the tumor type, the stage of neoplasia, the cellular context, as well as on the metabolic context in which the cells lie. Earliest reports suggested a role of autophagy against tumorigenesis favoring tumor cell death in an apoptotic-dependent or independent way (Levine and Yuan, 2005; Pattingre et al., 2005). In this regard, uncontrolled autophagy can lead to excessive degradation of cellular constituents and organelles required for the cells' homeostasis, and its enhancement represent a valid therapeutic target of many anticancer drugs. Hence, autophagy has been widely known as a tumor-suppressive mechanism and cancer cells with limited autophagy may suffer from increased oxidative stress, DNA damage, and genomic instability, which lead to the acquisition of genome mutations that drive tumorigenesis (Mathew et al., 2007; Mathew et al., 2009). On the other hand, autophagy also plays a protective role in cancer cells by removing damaged organelles or recycling misfolded macromolecules. Several studies report that autophagy tries to satisfy the high metabolic demands of the proliferating tumor cells suffering stressful conditions, such as nutrient deprivation, oxidative stress, hypoxia, or in response to therapy (Condo et al., 2005).

Hence, the possibility to modulate autophagy may represent a useful therapeutic approach to treat different types of cancer. In this regard, a variety of clinical trials based on autophagy modulators are ongoing. For instance, in tumors with enhanced autophagy, which acts as a survival mechanism and chemoresistance, its inhibition can make them more prone to initiate cell death mechanisms. In this sense, there are a variety of examples showing that autophagy inhibitors, as 3-methyl-adenine (3-MA) or hydroxychloroquine (HCQ), when used in combination with anticancer drugs, may sensitize chemoresistant cells, thus leading to cancer cell death (Jain et al., 2013; Lee et al., 2015). However, excessive autophagy induction upon cytotoxic drug treatments or through the use of autophagy inducers, as mTOR inhibitors, may also lead to autophagic cell death (Bursch et al., 1996; Tian et al., 2019).

On the other hand, recent advances in nanomedicine make it possible to fight cancer with effective therapeutic compounds, removing the obstacles encountered with traditional drugs. Interestingly, nanomaterials have been explored as potent modulators of autophagy through multiple mechanisms and have been exploited as therapeutic agents against cancer (Cordani and Somoza, 2019). For this reason, autophagy modulation with chemotherapy drugs, including nanoparticle-based strategies, would acquire clinical relevance in the near future, as a complementary therapy for the treatment of cancers.

This Research Topic gathers original research and review papers on novel drugs and nanomaterials in cancer treatment based on autophagy modulation and the related molecular mechanisms of action ranging from basic research to more applied translational and clinical studies. The 17 accepted articles consist of 9 Original Research articles and 8 Reviews or Mini-Reviews, demonstrating that inhibition or enhancement of the autophagy pathway may serve as an effective tool to counteract cancer.

Some of the research articles published describe the anticancer proprieties, both *in vivo* and *in vitro,* of several natural substances by modulating signaling pathways regulating autophagy in various tumors, thus representing great candidates for further therapeutic applications. Wang et al. showed that Sotetsuflavone, a naturally derived and occurring flavonoid, induced autophagy in lung cancer blocking PI3K/Akt/mTOR signaling pathway *in vivo* and *in vitro*, was able to increase the levels of pro-apoptotic markers, as cytochrome C or cleaved-caspase 3, and to reduce the expression of cyclin D1 and CDK4, thus blocking cell cycle in the G0/G1 phases. Tsai et al. reported that 6-Gingerol, a natural phenol found in ginger, inhibits lung cancer cell growth via suppression of USP14 expression and enhances autophagy-dependent ferroptosis, revealing the therapeutic efficacy of 6-Gingerol in A549 and its possible mechanism of action.

Natural compounds derived from herbal medicines have been extensively studied for their anticancer proprieties through modulation of signaling pathways leading to tumor cell death in various cancer types. In this context, Wu et *colleagues* showed that honokiol and magnolol, two compounds derived by magnolia bark extract, display therapeutic potential causing cell cycle arrest, induction of caspase 3-dependent apoptosis, and autophagy in high-grade urothelial carcinoma. Chen et al. explored the anti-breast cancer potential of ethanol crude extracts from Brucea javanica seed (BJE). Their data showed that BJE could inhibit breast cancer cell proliferation and induce apoptosis, together with the inhibition of autophagy, as demonstrated by the reduction of several autophagy-related markers and the increase of mTOR phosphorylation. Gao et al. revealed the molecular mechanisms through, which Moracin N (MAN), a secondary metabolite extracted from the leaves of Morus alba L, inhibited cell proliferation and induced apoptosis in lung cancer cells. Interestingly, they found that MAN treatment dysregulated mitochondrial function and enhanced autophagy flux, concomitantly to the inhibition of mTOR signaling pathway in a time- and dose-dependent manner.

Defects in autophagy lead to the accumulation of impaired mitochondria, which are a potential source of ROS that lead to DNA damage and genomic instability. In this regard, Chicote et al. reported that autophagy inhibitor 3-methyladenine displayed cytotoxicity in mouse fibroblasts and correlates with massive DNA damage, suggesting that in growing conditions, autophagy acts as a protective mechanism to diminish the intrinsic cytotoxicity of 3-methyladenine.

Recently, it has been shown that platinum compounds exert long-term effects, including autophagy activation in rat B50 neuroblastoma cells (Grimaldi et al., 2019). However, although cancer therapy based on platinum compounds display a potent anticancer effect, it also causes many side effects that limit its applicability. In this regard, Zhang et al. showed here that drug-induced platinum accumulation might also interfere with immune cells and, thus, increase the risk of immune toxicity in cancer patients.

However, due to their ability to elicit autophagy, metal-based compounds remain attractive agents for anticancer applications. Here, Chen et al. showed that nickel complex NiPT, a proteasomal inhibitor, was able to induce autophagy by dysregulating AMPK/mTOR signaling pathways in both cultured tumor cell lines and cancer cells derived from acute myeloid leukemia human patients. Interestingly, they found that NiPT leads to apoptosis in lung cancer cells if it is concomitantly used together with autophagy inhibitors, suggesting that NiPT-induced autophagy protects cancer cells from death.

The Research Topic also counts with several well-structured review articles that summarize studies describing novel drugs and nanomaterials in cancer treatment based on autophagy modulation and the related molecular mechanisms.

In recent years, several nanocarriers have been developed and investigated to improve the solubility, bioavailability, controlled release of therapeutics, and increase their cytotoxic effect on cancer cells. Sharma et al. highlighted that the use of nanoparticles for the delivery of miRNA-34a in cancer cells might inhibit critical autophagy-related pathways, thus sensitizing cancer cells to traditional chemotherapy drugs. On the other side, Condello et al. focused on the most effective liposomal formulations for the simultaneous transport of chemotherapeutics and autophagy inhibitors to control pharmacokinetics and targeting, thus reducing the adverse effects of drugs.

Alkylphosphocholine (APC) derivatives represent a novel class of antineoplastic agents that inhibit the serine–threonine kinase Akt, which acts as the primary regulator of cell survival. Berger *et colleagues* focused their attention on the capability of such structures to induce autophagy by inhibiting the Akt/mTOR cascade when administered as monotherapy or in combination with other drugs.

The cytoprotective role that autophagy plays under certain conditions during cancer therapy is well assumed, and many studies have been addressed to reduce protective autophagy to overcome drug resistance in the clinical practice. Liu et al. discussed the advantages of combining agents that induce cytoprotective autophagy with autophagy inhibitors as a cancer therapeutic approach in clinical application. Hence, the exploitation of appropriate autophagy might cooperate with traditional anticancer drugs to overcome drug resistance.

Iron-dependent ferroptosis is a type of cell death discovered in recent years, which is driven by lipid peroxidation and, recently, it has been linked with various kinds of diseases, including cancer. Lin et al. elegantly described the relationship between ferroptosis and autophagy and focused their interest on small molecules inducing ferroptosis, which have been observed to lead to a robust inhibitory effect on tumor growth and enhance the sensitivity to chemotherapeutic drugs. The tripartite motif family of proteins (TRIMs) has emerged as a critical regulator of the autophagy process, and their dysregulation is strongly connected to oncogenesis or cancer progression. The review of Mandell et al. covers how the actions of TRIM proteins intersect with and orchestrate autophagy, thus contributing to cancer progression and the possibility of targeting TRIM-directed autophagy in cancer therapy.

Recently, the interest in metabolic therapy for cancer has been renewed, particularly in amino acid deprivation by enzymes. In this context, L-asparaginase was approved for the treatment of acute lymphoblastic leukemia by the U.S. Food and Drug Administration (Truelove et al., 2013). Importantly, the combination of amino acid degrading enzymes and autophagy regulators has been demonstrated to show synergistic anticancer effects in preclinical and clinical studies (Song et al., 2015). In this sense, Wang et al. highlighted recent advances of several potential anticancer enzymes and the concomitant employment of autophagy modulators with amino acid degrading enzymes as potential cancer therapeutic approaches.

Recent studies have elucidated that autophagy, in addition to lead severe metabolic changes and chemoresistance also plays a role in regulating the immune system, and increasing attention has been focused on the potential applications of autophagy modulation for tumor immunotherapy. In this regard, Lim and Murthy summarized their current understanding of how autophagy drives tumor progression and chemoresistance via immunosuppression and they resumed an increasing set of pharmacologically actionable targets in the autophagy pathway, which may serve for novel cancer immunotherapies.

In conclusion, this collection of Review and Original Research articles critically summarizes the current understanding of how autophagy modulation could be exploited for cancer treatment. We believe that these advances will inspire basic and clinical scientists working in related fields to investigate molecular mechanisms and translational studies further.
